# Combining PD-1 or PD-L1 inhibitors with chemotherapy is a good strategy for the treatment of extensive small cell lung cancer: A retrospective analysis of clinical studies

**DOI:** 10.3389/fimmu.2022.1059557

**Published:** 2022-12-05

**Authors:** Hao Luo, Guangbin Song, Dong Wang, Mengxia Li, Nan Dai

**Affiliations:** ^1^ Cancer Center, Daping Hospital, Army Medical University, Chongqing, China; ^2^ College of Bioengineering, Key Lab of Biorheological Science and Technology, Ministry of Education, Chongqing University, Chongqing, China

**Keywords:** PD-L1 inhibitors, PD-1 inhibitors, extensive-stage small-cell lung cancer, chemotherapy, meta-analysis

## Abstract

**Objectives:**

To provide an updated systematic review and meta-analysis of published randomized controlled trials (RCTs) of the efficacy and safety of programmed cell death 1 (PD-1)/programmed cell death ligand 1 (PD-L1) inhibitors combined with chemotherapy versus chemotherapy alone in the treatment of extensive-stage small-cell lung cancer (ES-SCLC).

**Methods:**

PubMed, Web of Science, Embase, Clinicaltrials and the Cochrane Library were systematically searched to extract RCTs concerning the efficacy and safety of PD-1/PD-L1 inhibitors combined with chemotherapy versus chemotherapy alone in the treatment of ES-SCLC from the time of database inception to October 31, 2022. The literature was independently selected, information was extracted and the risk of bias of the RCTs was evaluated according to the inclusion and exclusion criteria. Stata14.0 was used for the meta-analysis.

**Results:**

Six studies involving 2,600 patients were included in the analysis. The results of the meta-analysis showed that the combination of PD-1/PD-L1 inhibitors significantly improved the OS (HR: 0.73, 95% CI: 0.66-0.80; *P*<0.0001), prolonged PFS (HR: 0.66,95% CI: 0.55-0.79; *P*<0.0001) and did not increase overall incidence of treatment-related adverse events (TRAEs) (RR: 1.03, 95% CI: 0.97-1.09; *P*=0.330) in ES-SCLC patients compared with chemotherapy alone. The subgroup analysis found that patients with negative PD-L1 expression (< 1%) benefited in OS, whereas patients with positive PD-L1 expression (≥1%) had no statistically significant difference in OS. There was a statistically significant difference in PFS between PD-L1-negative (< 1%) and PD-L1-positive (≥1%) patients. The addition of a PD-1 inhibitor or PD-L1 inhibitor to the chemotherapy regimen can improve OS and prolong PFS in patients with ES-SCLC.

**Conclusions:**

PD-1/PD-L1 inhibitors combination chemotherapy significantly improves PFS and OS in ES-SCLC patients without increasing the overall incidence of TRAEs.

## 1 Introduction

Small cell lung cancer (SCLC) is a type of tumour that exhibits fast growth, early metastasis and poor prognosis, and it accounts for approximately 15% of lung cancers (1, 2). Approximately 70% of SCLC patients are already in the extensive stage at the time of initial diagnosis (3). Chemotherapy is still the main treatment for extensive-stage small-cell lung cancer (ES-SCLC). Etoposide combined with platinum is the standard first-line chemotherapy regimen for ES-SCLC (4). However, the 5-year survival rate is less than 2% (5). Therefore, it is particularly important to identify new treatments to improve the survival rate of patients with ES-SCLC.

The advent of immunotherapy has resulted in new treatment options for improving the survival rate of ES-SCLC patients (6). Immune checkpoint inhibitors (ICIs) have achieved considerable breakthroughs in the treatment of many cancers (7–10). ICIs can block the negative costimulatory signalling pathway of T cells, thereby improving the body’s antitumour immune response and promoting the clearance of T cells to tumour cells (11, 12). At present, ICIs that are mainly used in clinical practice include cytotoxic T-lymphocyte associated protein 4 (CTLA-4) inhibitors, programmed cell death 1 (PD-1) inhibitors and programmed cell death ligand 1 (PD-L1) inhibitors (13). ICIs targeting CTLA-4 and PD-1/PD-L1 can block the immune checkpoint pathway to restore the body’s antitumour immune response and exert antitumour effects.

Among immunotherapy studies in ES-SCLC, clinical studies represented by Impower 133 and CASPIAN have achieved significant breakthroughs in the field of first-line treatments of ES-SCLC, with patients achieving unprecedented improvements in survival (14, 15). In 2022, the latest results of the ASTRUM-005 study showed that domestic PD-1 inhibitors combined with chemotherapy had a significant survival benefit in the first-line treatment of ES-SCLC (16). Moreover, PD-1/PD-L1 inhibitors combined with chemotherapy have become the new standard first-line treatment for ES-SCLC (3).

In recent years, several meta-analyses have evaluated the efficacy and safety of PD-1/PD-L1 inhibitors in combination with chemotherapy compared with chemotherapy alone for ES-SCLC (17–19). However, all of the randomized controlled trials (RCTs) that were included in these meta-analyses were conducted before 2020. IMpower133 and CASPIAN updated the data from the trials in 2021 and 2022, respectively (14, 15). In addition, two more important trials were published in 2022 (ASTRUM-005 and CAPSTONE-1) (16, 20). Therefore, there is a strong need for an updated meta-analysis of RCTs of PD-1/PD-L1 inhibitor combination chemotherapy versus chemotherapy alone for ES-SCLC to provide evidence for supporting the development of clinical practice guidelines.

## 2 Methods

### 2.1 Inclusion and exclusion criteria

#### 2.1.1 Type of study

RCTs of PD-1/PD-L1 inhibitors combined with chemotherapy versus chemotherapy alone in the treatment of ES-SCLC were included in this study.

#### 2.1.2 Participants

Patients with SCLC confirmed by histopathology and/or cytology were included in this study.

#### 2.1.3 Interventions

The experimental group received PD-1/PD-L1 inhibitors combined with chemotherapy. The control group received chemotherapy alone.

#### 2.1.4 Outcomes

1. Overall survival (OS).
2. Progression-free survival (PFS).
3. Overall incidence of treatment-related adverse events (TRAEs).

### 2.2 Exclusion criteria

1. Duplicate publications from the literature.
2. Data that could not be extracted from the literature.
3. Non-RCTs.

### 2.3 Search strategy

PubMed, Web of Science, Embase, ClinicalTrials.gov and the Cochrane Library were systematically searched to extract RCTs of the efficacy and safety of PD-1/PD-L1 inhibitors combined with chemotherapy versus chemotherapy alone in the treatment of ES-SCLC from the time of database inception to October 31, 2022.

### 2.4 Data extraction

Two evaluators read the title, abstract or full text to identify the literature that met the inclusion criteria, and they also cross-checked the results of the included trials. In cases of disagreement, the third evaluator decided whether to include the study or not. Information was extracted by using a predeveloped homemade literature characteristics table. The extracted information included the name of the trial, year of publication, authors, trial conduct time duration, sample size, age, sex, dosing regimen, follow-up time and outcome indicators.

### 2.5 Quality assessment

The inclusion of RCTs was performed in strict accordance with the “Risk of bias Assessment method” recommended by the Cochrane Handbook (21). The evaluation included random assignment scheme generation, concealed grouping, blinding of performers and participants, blinding of outcome evaluators, incomplete outcome data, selected outcome reporting and other biases. “Low risk”, “unclear” and “high risk” were each evaluated separately.

### 2.6 Data analysis

A meta-analysis was performed by using Stata 14.0. Dichotomous variables were expressed as relative risks (RRs), and 95% confidence intervals (CIs) were calculated. Hazard ratios (HRs) and 95% CIs were collected to estimate the pooled estimates for survival outcomes (OS and PFS). The χ2 test was used to analyse the heterogeneity among the included RCTs. If P≥0.1 and I^2^<50%, a fixed-effects model was used; otherwise, if P<0.1 and I^2^≥50%, a random-effects model was used. A subgroup analysis was performed to determine the prediction of the immune response according to the expression of PD-L1 (< 1% PD-L1 vs. ≥1% PD-L1) and types of ICIs (PD-1 inhibitor plus chemotherapy vs. PD-L1 inhibitor plus chemotherapy). A sensitivity analysis was performed on OS to test the stability of the meta-analysis results. If no less than 10 papers were included, a publication bias analysis was performed by using funnel plots (22).

## 3 Results

### 3.1 Literature screening results

**Figure 1:**
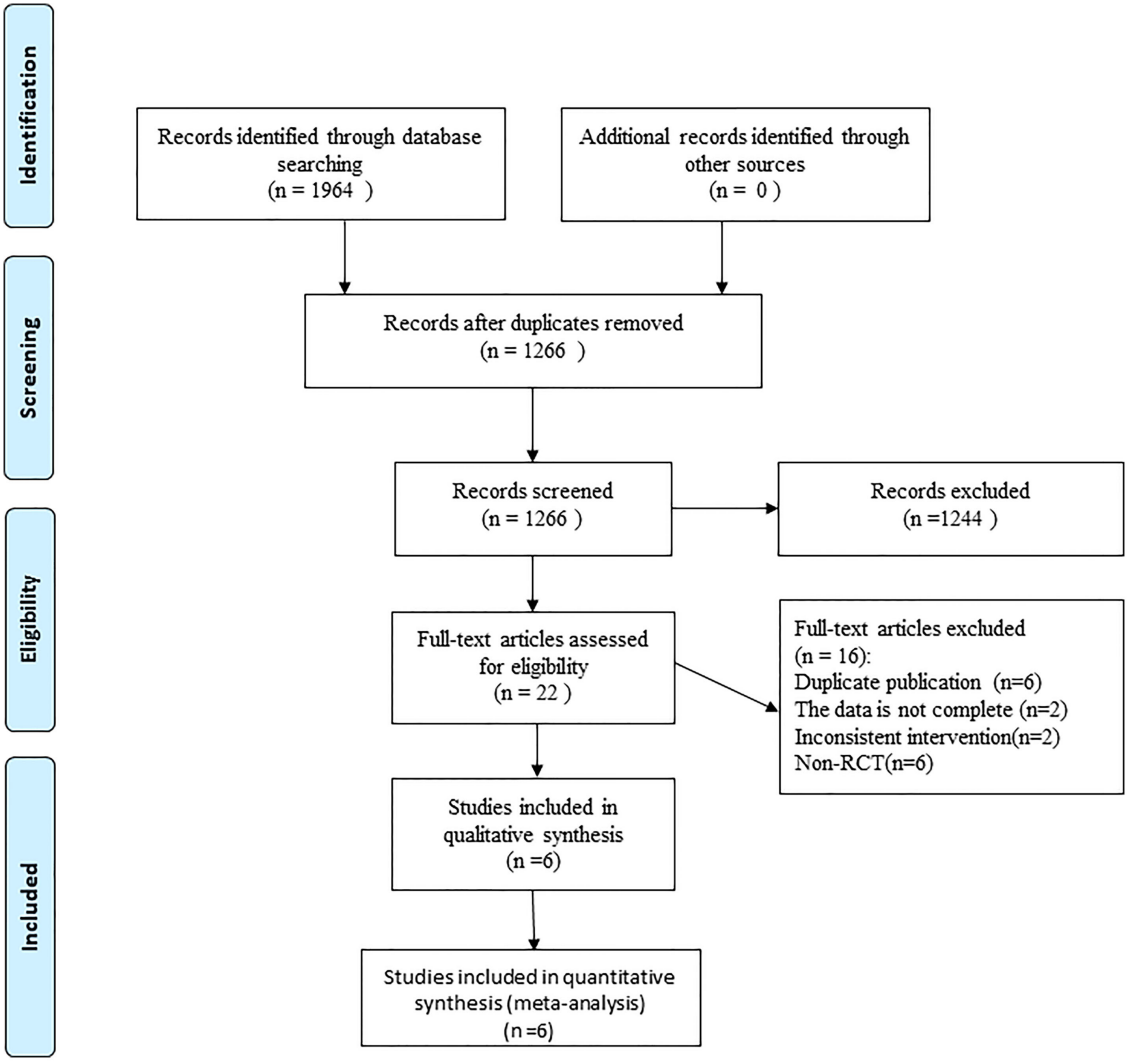
Flow chart of trial selection process.

A total of 1,964 articles were detected according to the search strategy. After eliminating the duplicate references *via* EndNote, 1,266 papers remained for the analysis. Subsequently, we read the title and abstract according to the PICO principle and excluded 1,244 articles. After reading the full text, 6 articles (14–16, 20, 23, 24) were finally included in the analysis. The literature retrieval process is shown in **Figure 1**.

### 3.2 Characteristics of the included literature

A total of 6 high-quality RCTs (14–16, 20, 23, 24) were included. ASTRUM-005 and CAPSTONE-1 are newly published studies in 2022. Two studies included updated data for IMpower133 and CASPIAN. The immunosuppressants that were involved were serplulimab, adebrelimab, durvalumab, atezolizumab, nivolumab and pembrolizumab. The characteristics of the included RCTs are summarized in **Table 1**.

**Table 1:** Characteristics of the included trials.

Trial name	Study (year)	Trial registration number	Phase	Time of trial	Patients (n)	Age<65/≥65		Male/female		ECOG PS (0/1)		Brain metastases(Yes/No)		Therapeutic schedule		Follow-up(month s)
						T	C	T	C	T	C	T	C	T	C	
ASTRUM-005	Cheng et al 2022	NCT04063163	III	September 12, 2019~April 27, 2021	585(389/196)	235/154	119/77	317/72	164/32	71/318	32/164	50/339	28/168	Carboplatin+etoposide+ serplulimab (PD-1 inhibitors)	Carboplatin+etoposide+placebo	12.3
CAPSTONE-1	Wang et al 2022	NCT03711305	III	December 26, 2018~ September 4, 2020	462(230/232)	155/75	147/85	184/46	188/44	33/197	30/202	5/225	5/227	Carboplatin+etoposide+adebrelimab(PD-L1 inhibitors)	Carboplatin+etoposide+placebo	13.5
CASPIAN	Paz-Ares et al 2022	NCT03043872	III	March 27, 2017~ March 22, 2021	537(268/269)	167/101	157/112	190/78	184/85	99/169	90/179	28/240	27/242	Cisplatin or carboplatin+durvalumab(PD-L1 inhibitors)	Cisplatin or carboplatin	39.4
IMpower133	Liu et al 2021	NCT02763579	III	June 7, 2016~January 24, 2019	403(201/202)	111/90	106/96	129/72	132/70	73/128	67/135	17/184	18/184	Carboplatin+ etoposide+atezolizumab(PD-L1 inhibitors)	Carboplatin+ etoposide+placebo	22.9
EA5161	Leal et al 2020	NCT03382561	II	May 2018~December 2018	160(80/80)	NR	NR	35/45	36/44	23/57	24/56	NR	NR	Cisplatin or carboplatin+nivolumab(PD-1 inhibitors)	Cisplatin or carboplatin	NR
KEYNOTE-604	Rudin et al 2020	NCT03066778	III	May 15, 2017~ July 30, 2018	453(228/225)	115/113	101/124	152/76	142/83	60/168	56/169	33/195	22/203	Platinum+etoposide + pembrolizumab(PD-1 inhibitors)	Platinum+etoposide + placebo	21.6

NR, Not reported; T,treatment group; C, control group.

### 3.3 Risk of bias

**Figure 2:**
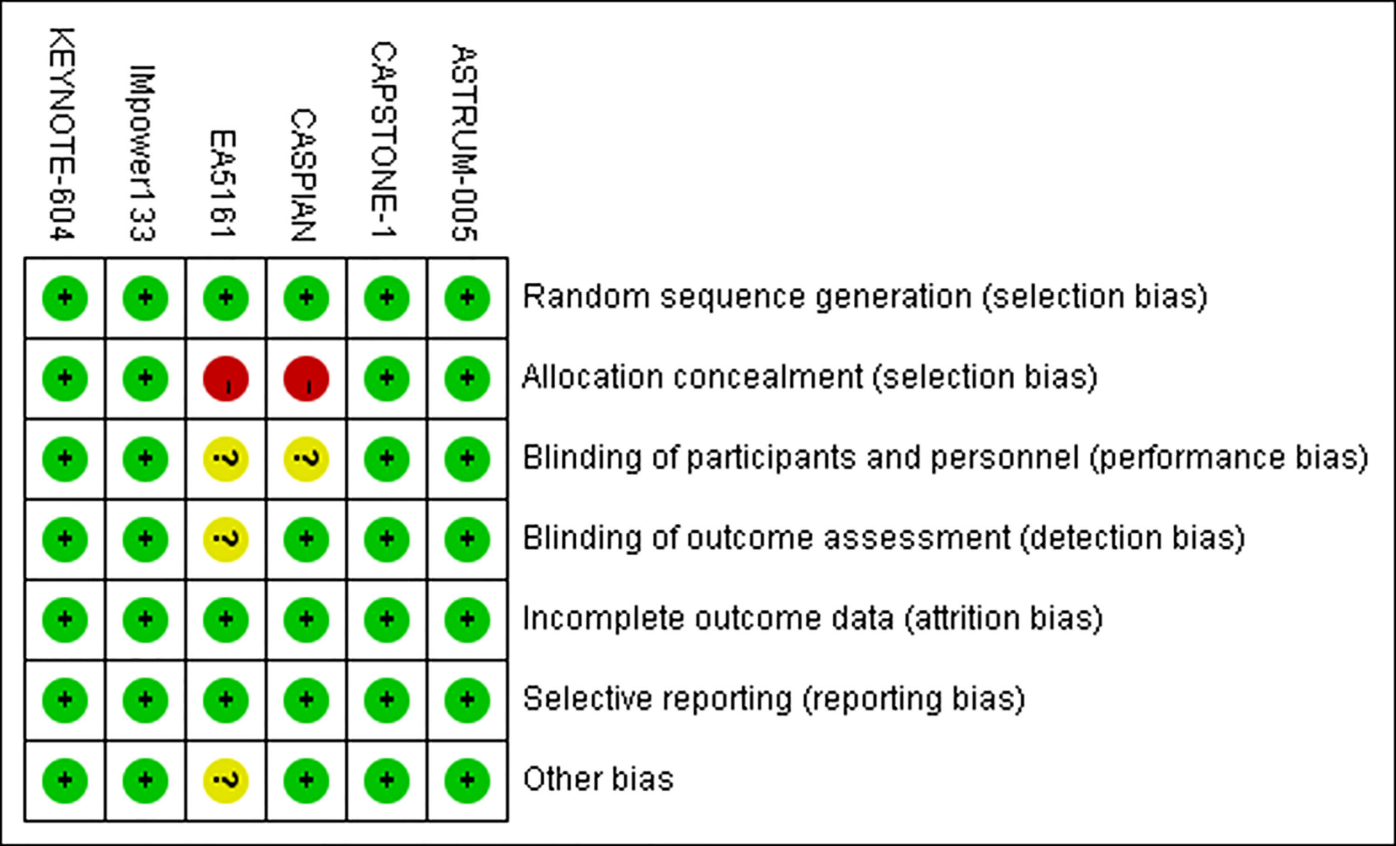
Risk of bias summary.

Two studies exhibited high risks of bias for the allocation of concealed entries. Blinding entries for investigators and patients were unclear in 2 studies. Additionally, one study showed unclear entries for blinding of outcome measures and other sources of bias. The remaining 4 studies exhibited low risks of bias for each entry evaluation. The risk of bias assessment is shown in **Figure 2**.

### 3.4 Overall survival

**Figure 3:**
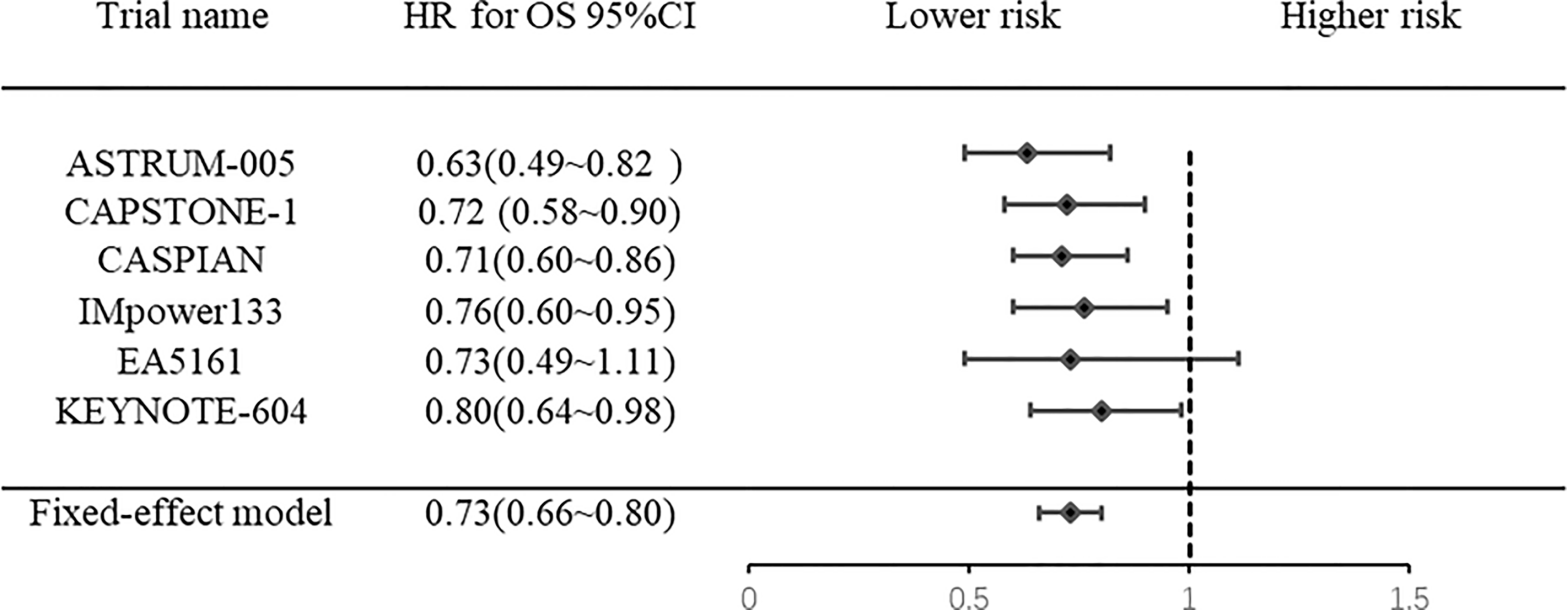
Forest plot of meta-analysis for overall survival.

Six RCTs were included without heterogeneity (*I^2 =^* 0%, *P*= 0.824). The pooled results showed that PD-1/PD-L1 inhibitor combination chemotherapy significantly improved OS (HR: 0.73, 95% CI: 0.66-0.80; *P*<0.0001) (**Figure 3**) in ES-SCLC patients compared to chemotherapy alone.

### 3.5 Progression-free survival

**Figure 4:**
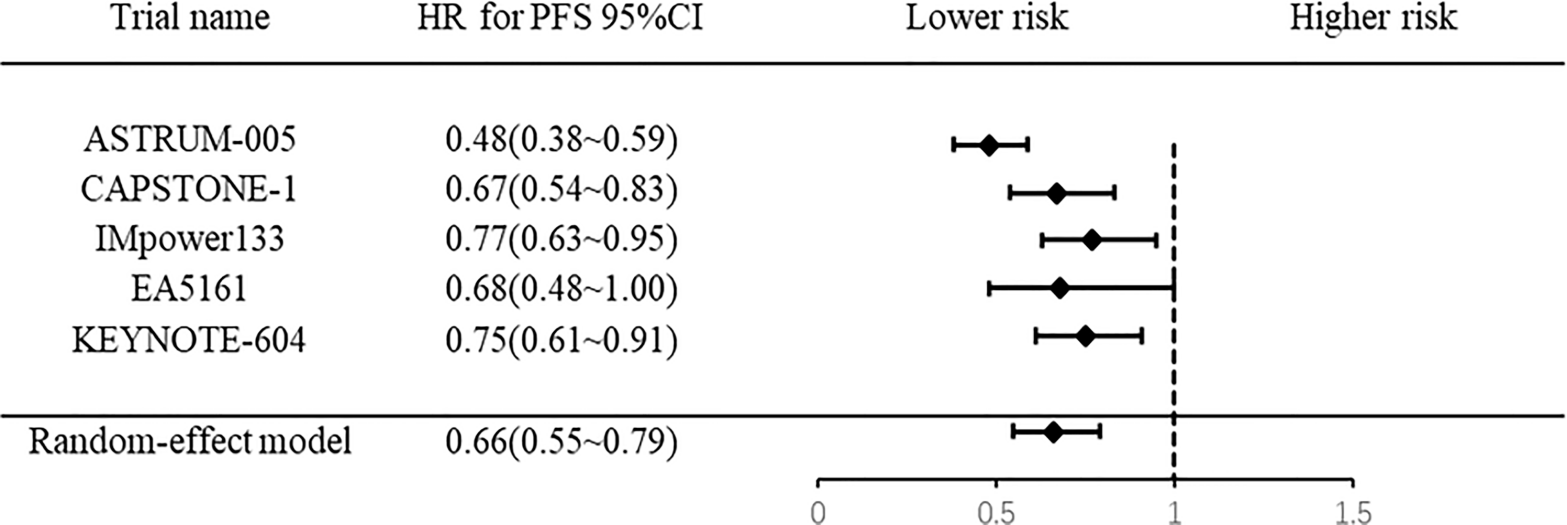
Forest plot of meta-analysis for progression free survival.

Five RCTs were included, and they exhibited heterogeneity *(I^2 =^* 66.1%, *P*=0.019). The pooled results showed that PD-1/PD-L1 inhibitor combination chemotherapy significantly improved PFS (HR: 0.66, 95% CI: 0.55-0.79; *P*<0.0001) (**Figure 4**) in ES-SCLC patients compared to chemotherapy alone.

### 3.6 Overall incidence of treatment-related adverse events

**Figure 5:**
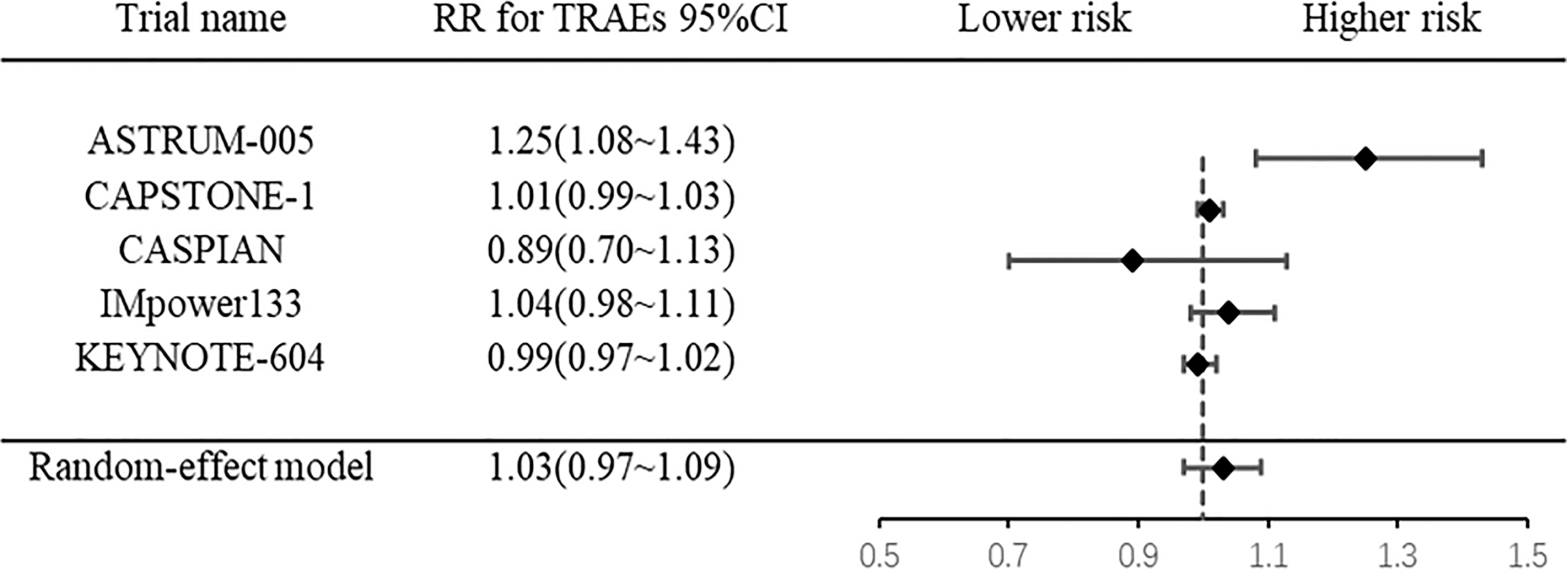
Forest plot of meta-analysis for overall incidence of treatment-related adverse events.

Five RCTs were included, and they exhibited heterogeneity (*I^2 =^* 87.7%, *P*<0.0001). The pooled results showed that there was no difference in the overall incidence of TRAEs between chemotherapy alone and PD-1/PD-L1 inhibitor plus chemotherapy (RR: 1.03, 95% CI: 0.97-1.09; *P*= 0.330) (**Figure 5**).

### 3.7 Subgroup analysis

**Figure 6:**
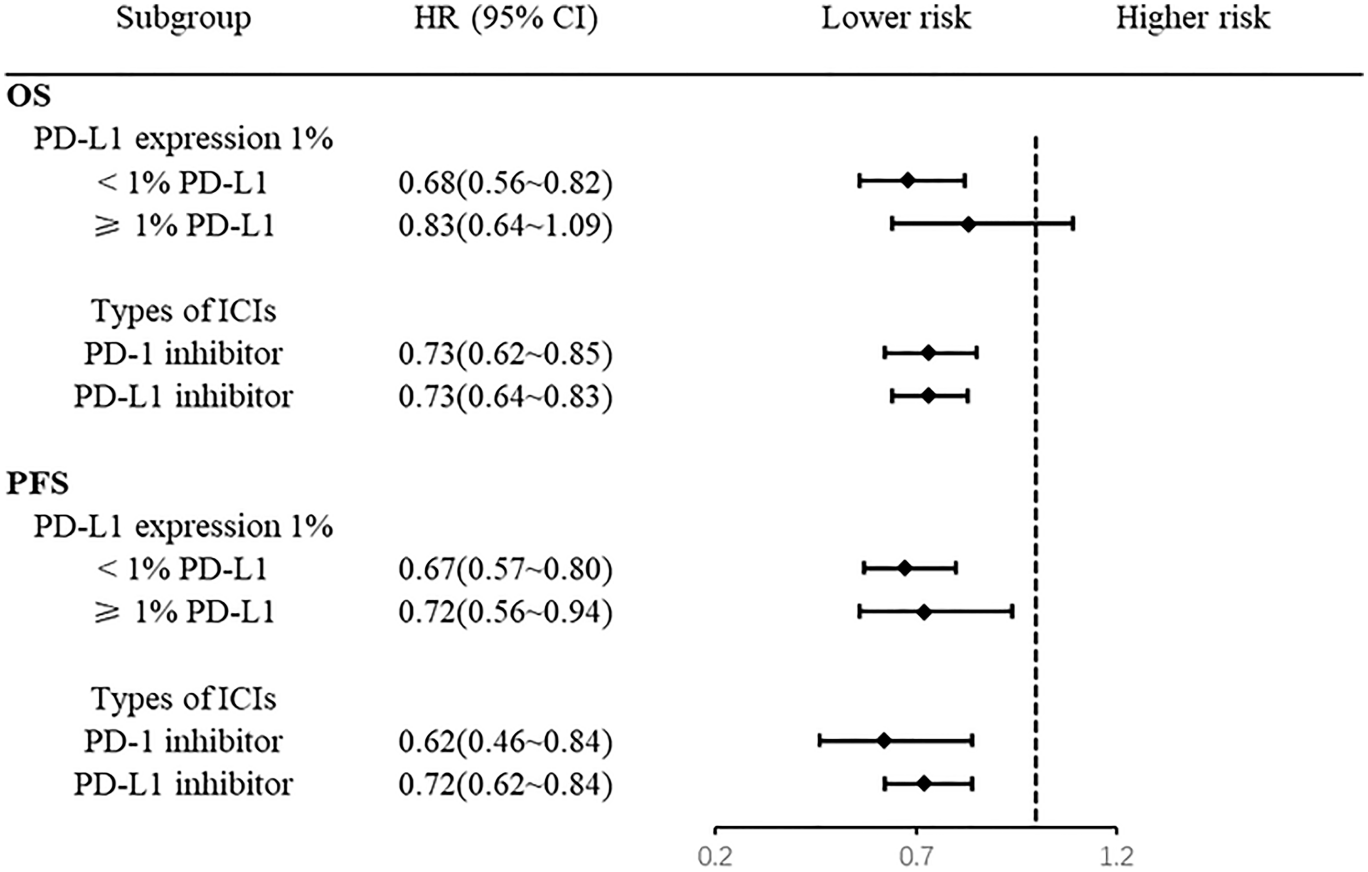
Forest plot of subgroup analysis.

Patients with negative PD-L1 expression (< 1%) benefited from OS, whereas patients with positive PD-L1 expression (≥1%) had no significant difference in OS (**Figure 6**). There was a significant difference in PFS between the PD-L1-negative (< 1%) and PD-L1-positive (≥1%) groups (**Figure 6**). The addition of a PD-1 inhibitor or PD-L1 inhibitor to the chemotherapy regimen improved OS and prolonged PFS in patients with ES-SCLC (**Figure 6**).

### 3.6 Sensitivity analysis

We performed a sensitivity analysis on OS. The included studies were excluded one by one, and the results did not significantly change, thus suggesting a low sensitivity and more robust and reliable results.

### 3.7 Publication bias

As fewer than 10 studies were included, publication bias detection could not be performed.

## 4 Discussion

ES-SCLC is a common clinical subtype of lung cancer that has a poor prognosis, short survival period and high disease burden due to limited treatment options and easy drug tolerance (25–27). In the last 30 years, there has been no significant breakthrough in the treatment of ES-SCLC, and the overall prognosis has not significantly improved. With the advent of the era of immunotherapy, ICIs have made significant progress in the treatment of ES-SCLC (12, 28). The emergence of ICIs has provided new treatment options for ES-SCLC patients (29). Additionally, the U.S. Food and Drug Administration has approved carboplatin and etoposide combined with the PD-L1 inhibitor atezolizumab as a first-line therapy, as well as the single-agent PD-1 inhibitors nivolumab and pembrolizumab as a third-line therapy (3, 30). In 2018, the IMpower133 study evaluated the efficacy of atezolizumab plus chemotherapy (31). The results showed that the median OS was improved and that the median PFS was prolonged in the chemotherapy plus atezolizumab group. Subsequent studies with updated data still suggested a benefit of PD-L1 inhibitors combined with chemotherapy in the first-line treatment of ES-SCLC. The KEYNOTE604 study evaluated the efficacy of pembrolizumab plus chemotherapy and found that the pembrolizumab plus chemotherapy group had improved median OS and significantly prolonged median PFS compared with the control group (24). The latest results from the ASTRUM-005 study showed that the PD-1 inhibitor serplulimab combined with chemotherapy has a significant survival benefit and a good safety profile in the first-line treatment of ES-SCLC (16). The results of the abovementioned series confirm that combining PD-1 inhibitors or PD-L1 inhibitors with chemotherapy is a more successful treatment strategy for patients with ES-SCLC. Several previous meta-analyses have also supported the significant survival benefit of PD-1/PD-L1 inhibitors in combination with chemotherapy for ES-SCLC (17–19). The present study included six internationally renowned clinical trials in an updated meta-analysis based on previous work. The results of the meta-analysis showed that PD-1/PD-L1 inhibitors combined with chemotherapy significantly improved OS and PFS in ES-SCLC patients. In terms of the overall incidence of TRAEs, there was no significant difference between PD-1/PD-L1 inhibitor combination chemotherapy and chemotherapy alone.

The selection of predictive markers for assessing the efficacy of immunotherapy in ES-SCLC is of clinical significance. PD-L1 is a tumour cell surface molecule, and its use as a biomarker for immunotherapy has been widely used in various cancer types (32, 33). Therefore, it is hypothesized that SCLC patients with high PD-L1 expression may benefit from ICI treatment. With the development of immunotherapy studies in SCLC, the feasibility of PD-L1 as a marker of SCLC efficacy has also received considerable attention. The proportion of PD-L1-positive tumour cells in SCLC is low and accounts for approximately 18-32% (34, 35). This result suggests that most ES-SCLC patients do not benefit from immunotherapy. The characteristics of long-term SCLC survivors that were reported in the IMpower133 study found no significant correlation between PD-L1 expression levels and long-term survival benefits from immunotherapy (15). The KEYNOTE-604 study and the CASPIAN study obtained similar conclusions (14, 24). Therefore, the results of existing clinical trials do not yet support PD-L1 expression as a biomarker of immune efficacy in SCLC. However, we found that patients with negative PD-L1 expression benefited from OS according to a subgroup analysis. This finding indicates that PD-L1 expression is correlated with the OS of ES-SCLC, and patients who are negative for PD-L1 can benefit from immunotherapy. However, this is in stark contrast to the current widely held viewpoint that patients with tumours with high PD-L1 expression who receive ICIs can benefit from OS (33, 36). First, the different criteria for positive PD-L1 expression in the subgroup analyses may have contributed to this result. Second, unlike non-small cell lung cancer, PD-L1 is mainly expressed in tumour-infiltrating immune cells rather than tumour cells in SCLC (17). Third, there are four genetic subtypes of SCLC, among which SCLC-I has a good response to immunotherapy (37). Therefore, different SCLC types also influence prognoses. These clinical studies require a more detailed classification of SCLC. Fourth, it may be related to tumour immune escape mediated by exosomal PD-L1 (38). In addition to their own high expression of PD-L1 to suppress immune system-mediated immune evasion, tumour cells can also release PD-L1-carrying exosomes that are equally capable of remotely interfering with immune cell activity (39, 40). These reasons can explain why ES-SCLC patients with negative PD-L1 expression can benefit from OS. However, this conclusion requires prospective studies to evaluate the predictive value of PD-L1 expression in ES-SCLC immunotherapy. Circulating tumour cells (CTCs) are a “liquid biopsy specimen” that can replace the primary tumour (41). The expression of PD-L1 in CTCs can be used to evaluate the efficacy of PD-1/PD-L1 mAbs in non-small cell lung cancer patients (42, 43). The use of CTCs to evaluate the expression of PD-L1 in SCLC may overcome the heterogeneity of PD-L1 expression.

According to the current clinical trials, both PD-1 inhibitors combined with chemotherapy and PD-L1 inhibitors combined with chemotherapy can benefit the survival rate of patients with ES-SCLC. The idea of whether PD-1 inhibitors differ from PD-L1 inhibitors in clinical outcomes is controversial. Yu et al. (17) conducted a meta-analysis of the efficacy and safety of PD-L1 inhibitors versus PD-1 inhibitors in first-line chemotherapy for ES-SCLC. The results showed that PD-L1 inhibitors combined with chemotherapy and PD-1 inhibitors combined with chemotherapy significantly prolonged the survival times of patients with ES-SCLC compared with chemotherapy alone. An indirect comparison showed no significant difference in clinical efficacy between PD-L1 inhibitors combined with chemotherapy and PD-1 inhibitors combined with chemotherapy. The subgroup analysis found that both PD-L1 inhibitors and PD-1 inhibitors improved the survival times of ES-SCLC patients. The 2022 NCCN Oncology Clinical Practice Guideline recommends combination chemotherapy with atezolizumab and duvalizumab as the first-line treatment for ES-SCLC. In the 2022 CSCO guidelines for the diagnosis and treatment of SCLC, both atezolizumab and duvalizumab are recommended as grade I preferred first-line treatments for patients with ES-SCLC. The results of this meta-analysis support the evidence-based results that SCLC guidelines recommend PD-L1 inhibitors as the first-line therapy for ES-SCLC. The phase III KEYNOTE-604 study showed that first-line pembrolizumab plus chemotherapy reduced the risk of disease progression in SCLC (4.5 months vs. 4.3 months), but there was no significant difference in OS (24). Therefore, in 2021, Merck Sharp & Dohme (MSD) voluntarily withdrew the indication for pembrolizumab for ES-SCLC. In addition, the EA5161 clinical study showed that PD-1 inhibitors combined with chemotherapy failed to improve OS in ES-SCLC (23). However, the latest edition of the CSCO guidelines in 2022 added a level III recommendation for the first-line treatment of ES-SCLC with serulizumab combined with chemotherapy. In the phase III study of ASTRUM-005, the median overall survival was 15.38 months in the silulimab group and 11.10 months in the placebo group (16). In addition, the 24-month overall survival rates were 43.1% and 7.9%, respectively. ASTRUM-005 resolved the limitation that immunotherapy in the previous IMpower133 and CASPIAN studies only resulted in a survival benefit of approximately 2 months in ES-SCLC. The success of the ASTRUM-005 study is the first breakthrough to achieve a significant improvement in OS outcome in ES-SCLC with a PD-1 inhibitor being used as a first-line therapy, thus providing a new option for the first-line treatment of ES-SCLC. This meta-analysis, which combined the results of a subgroup analysis of three clinical trials, showed that PD-1 inhibitors combined with chemotherapy could benefit the survival of patients with ES-SCLC. Subsequently, serplulimab is expected to move to a Tier I recommendation, thus replacing the use of PD-L1 inhibitors. According to relevant literature reports, PD-1 inhibitors may have better efficacy than PD-L1 inhibitors, and the overall incidence of adverse events is similar (44, 45). However, PD-1 inhibitors have a higher incidence of pneumonia (46, 47). Therefore, clinical application of these inhibitors should be based on the clinical study data and approved indications of different drugs for treatment selection.

Our study had some limitations. First, a publication bias analysis could not be performed due to the limited number of enrolments. Therefore, publication bias may have existed in this meta-analysis. Second, significant heterogeneity was observed in the analysis of the total incidence of PFS and TRAEs in this study, and different types, doses and administration frequencies of immunosuppressive agents and chemoradiotherapy may be sources of heterogeneity.

In conclusion, based on the current meta-analysis, PD-1/PD-L1 inhibitors combined with chemotherapy significantly improved PFS and OS in patients with ES-SCLC without increasing the overall incidence of TRAEs. Therefore, PD-1/PD-L1 inhibitors combined with chemotherapy can be used as the first-line treatment for patients with ES-SCLC.

## Data availability statement

The original contributions presented in the study are included in the article/supplementary material. Further inquiries can be directed to the corresponding authors.

## Author contributions

Conceptualization: HL and ND. Data collection: GS, ML and DW. Funding acquisition: ND. Resources: ND. Software: HL. Supervision: HL. Writing–original draft: HL and ND. Writing—review and editing: HL and ND. All authors listed have made a substantial, direct, and intellectual contribution to the work and approved it for publication.

## Funding

This work is supported by the Science and Technology Innovation Enhancement Project of Army Medical University (STIEP, 2018XLC3061 to ND) and Science and Technology Innovation Enhancement Project of Army Medical University (STIEP 2019XLC1013 to DW).

## Conflict of interest

The authors declare that the research was conducted in the absence of any commercial or financial relationships that could be construed as a potential conflict of interest.

## Publisher’s note

All claims expressed in this article are solely those of the authors and do not necessarily represent those of their affiliated organizations, or those of the publisher, the editors and the reviewers. Any product that may be evaluated in this article, or claim that may be made by its manufacturer, is not guaranteed or endorsed by the publisher.

## References

[1] Yuan M Zhao Y Arkenau HT Lao T Chu L Xu Q . Signal pathways and precision therapy of small-cell lung cancer. Signal transduction targeted Ther (2022) 7(1):187. doi: 10.1038/s41392-022-01013-y PMC9200817 35705538

[2] Luo H Shan J Zhang H Song G Li Q Xu CX . Targeting the epigenetic processes to enhance antitumor immunity in small cell lung cancer. Semin Cancer Biol (2022) 86(Pt 3):960–70. doi: 10.1016/j.semcancer.2022.02.018 35189321

[3] Wang Z Mai S Lv P Xu L Wang Y . Etoposide plus cisplatin chemotherapy improves the efficacy and safety of small cell lung cancer. Am J Trans Res (2021) 13(11):12825–33. PMC8661205 34956497

[4] Chen H Horita N Ito K Nagakura H Hara Y Kobayash N . Systematic review of first-line chemotherapy for chemo-naïve extensive-stage small-cell lung cancer: network meta-analysis. Ther Adv Med Oncol (2020) 12, 1–4. doi: 10.1177/1758835920965841 PMC7745559 33403010

[5] Yang S Zhang Z Wang Q . Emerging therapies for small cell lung cancer. J Hematol Oncol (2019) 12(1):47. doi: 10.1186/s13045-019-0736-3 31046803 PMC6498593

[6] Melosky B Cheema PK Brade A McLeod D Liu G Price PW . Prolonging survival: The role of immune checkpoint inhibitors in the treatment of extensive-stage small cell lung cancer. oncologist. (2020) 25(11):981–92. doi: 10.1634/theoncologist.2020-0193 PMC7648366 32860288

[7] Niglio SA Jia R Ji J . Programmed death-1 or programmed death ligand-1 blockade in patients with platinum-resistant metastatic urothelial cancer: A systematic review and meta-analysis. Eur urology. (2019) 76(6):782–9. doi: 10.1016/j.eururo.2019.05.037 31200951

[8] Vickers AD Winfree KB Cuyun Carter G . Relative efficacy of interventions in the treatment of second-line non-small cell lung cancer: a systematic review and network meta-analysis. BMC cancer. (2019) 19(1):353. doi: 10.1186/s12885-019-5569-5 30987609 PMC6466705

[9] Chen H Horita N Ito K Nagakura H Hara Y Kobayash N . Risk of pneumonitis and pneumonia associated with immune checkpoint inhibitors for solid tumors: A systematic review and meta-analysis. Front Immunol (2019) 10:108. doi: 10.3389/fimmu.2019.00108 30778352 PMC6369169

[10] Li Q Zhou ZW Lu J . PD-L1(P146R) is prognostic and a negative predictor of response to immunotherapy in gastric cancer. Mol Ther (2022) 30(2):621–31. doi: 10.1016/j.ymthe.2021.09.013 PMC8821936 34547468

[11] Rijavec E Genova C Biello F Rossi G Indini A Grossi F . Current state of the art and future perspectives with immunotherapy in the management of small cell lung cancer. Expert Rev Respir Med (2021) 15(11):1427–35. doi: 10.1080/17476348.2021.1987887 34590937

[12] Korde R Veluswamy R Allaire JC Barnes G . Small cell lung cancer patients treated with immune checkpoint inhibitor: A systematic literature review of treatment efficacy, safety and quality of life. Curr Med Res opinion. (2022) 38(8):1361–8. doi: 10.1080/03007995.2022.2078101 35575164

[13] Guo L Liang J Dai W Li J Si Y Ren W . PD-1/L1 with or without CTLA-4 inhibitors versus chemotherapy in advanced non-small cell lung cancer. Cancer control (2022) 29:1–11. doi: 10.1177/10732748221107590 PMC9185001 35673884

[14] Paz-Ares L Chen Y Reinmuth N Hotta K Trukhin D Statsenko G . Durvalumab, with or without tremelimumab, plus platinum-etoposide in first-line treatment of extensive-stage small-cell lung cancer: 3-year overall survival update from CASPIAN. ESMO Open (2022) 7(2):100408. doi: 10.1016/j.esmoop.2022.100408 35279527 PMC9161394

[15] Liu SV Reck M Mansfield AS Mok T Scherpereel A Reinmuth N . Updated overall survival and PD-L1 subgroup analysis of patients with extensive-stage small-cell lung cancer treated with atezolizumab, carboplatin, and etoposide (IMpower133). J Clin Oncol (2021) 39(6):619–30. doi: 10.1200/JCO.20.01055 PMC8078320 33439693

[16] Cheng Y Han L Wu L Chen J Sun H Wen G . Effect of first-line serplulimab vs placebo added to chemotherapy on survival in patients with extensive-stage small cell lung cancer: The ASTRUM-005 randomized clinical trial. Jama. (2022) 328(12):1223–32. doi: 10.1001/jama.2022.16464 PMC9516323 36166026

[17] Yu H Chen P Cai X Chen C Zhang X He L . Efficacy and safety of PD-L1 inhibitors versus PD-1 inhibitors in first-line treatment with chemotherapy for extensive-stage small-cell lung cancer. Cancer immunology immunotherapy: CII. (2022) 71(3):637–44. doi: 10.1007/s00262-021-03017-z PMC10992902 34297160

[18] Chen CY Chen WC Hung CM Wei YF . Chemotherapy or chemo-immunotherapy as first-line treatment for extensive-stage small-cell lung cancer: A meta-analysis. Immunotherapy. (2021) 13(14):1165–77. doi: 10.2217/imt-2021-0135 34261336

[19] Liu X Xing H Zhang H Liu H Chen J . Immunotherapy versus standard chemotherapy for treatment of extensive-stage small-cell lung cancer: a systematic review. Immunotherapy. (2021) 13(12):989–1000. doi: 10.2217/imt-2020-0284 34114477

[20] Wang J Zhou C Yao W Wang Q Min X Chen G . Adebrelimab or placebo plus carboplatin and etoposide as first-line treatment for extensive-stage small-cell lung cancer (CAPSTONE-1): A multicentre, randomised, double-blind, placebo-controlled, phase 3 trial. Lancet Oncol (2022) 23(6):739–47. doi: 10.1016/S1470-2045(22)00224-8 35576956

[21] Shuster JJ . Review: Cochrane handbook for systematic reviews for interventions, version 5.1.0, published 3/2011. Julian P.T. Higgins and s green. Res Synthesis Methods (2011) 2(2):126–30. doi: 10.4317/jced.59750

[22] Irwig L Macaskill P Berry G Glasziou P . Bias in meta-analysis detected by a simple, graphical test. graphical test is itself biased. BMJ (Clinical Res ed). (1998) 316(7129):470. PMC2665595 9492687

[23] Leal T Wang Y Dowlati A Lewis D Chen Y Mohindra AR . Randomized phase II clinical trial of cisplatin/carboplatin and etoposide (CE) alone or in combination with nivolumab as frontline therapy for extensive-stage small cell lung cancer (ES-SCLC): ECOG-ACRIN EA5161. J Clin Oncol (2020) 38(suppl 15):9000. doi: 10.1200/JCO.2020.38.15_suppl.9000 PMC12276847 40526078

[24] Rudin CM Awad MM Navarro A Gottfried M Peters S Csőszi T . Pembrolizumab or placebo plus etoposide and platinum as first-line therapy for extensive-stage small-cell lung cancer: Randomized, double-blind, phase III KEYNOTE-604 study. J Clin Oncol (2020) 38(21):2369–79. doi: 10.1200/JCO.20.00793 PMC7474472 32468956

[25] Bianco A D'Agnano V Matera MG Della Gravara L Perrotta F Rocco D . Immune checkpoint inhibitors: A new landscape for extensive stage small cell lung cancer treatment. Expert Rev Respir Med (2021) 15(11):1415–25. doi: 10.1080/17476348.2021.1964362 34374626

[26] Levy A Botticella A Le Péchoux C Faivre-Finn C . Thoracic radiotherapy in small cell lung cancer-a narrative review. Trans Lung Cancer Res (2021) 10(4):2059–70. doi: 10.21037/tlcr-20-305 PMC8107758 34012814

[27] Pacheco JM . Systemic therapy options following first-line chemoimmunotherapy in small-cell lung cancer. J Thorac disease. (2020) 12(10):6264–74. doi: 10.21037/jtd.2020.03.67 PMC7656348 33209465

[28] Xu Y Chen M Ding Y Guo F Chen M Lu T . The efficacy and safety of immune checkpoint inhibitor in patients with relapsed small-cell lung cancer: A systematic review and meta-analysis. J Clin Pharm Ther (2022) 47(4):421–9. doi: 10.1111/jcpt.13552 34734431

[29] Liu X Xing H Liu B . Current status and future perspectives of immune checkpoint inhibitors in extensive-stage small cell lung cancer. Am J Cancer Res (2022) 12(6):2447–64. PMC9251690 35812062

[30] Neumann M Murphy N Seetharamu N . The evolving role of PD-L1 inhibition in non-small cell lung cancer: A review of durvalumab and avelumab. Cancer Med J (2022) 5(1):31–45. 35253011 PMC8896901

[31] Horn L Mansfield AS Szczęsna A Havel L Krzakowski M Hochmair MJ . First-line atezolizumab plus chemotherapy in extensive-stage small-cell lung cancer. N Engl J Med (2018) 379(23):2220–9. doi: 10.1056/NEJMoa1809064 30280641

[32] Shi Y Lei Y Liu L Zhang S Wang W Zhao J . Integration of comprehensive genomic profiling, tumor mutational burden, and PD-L1 expression to identify novel biomarkers of immunotherapy in non-small cell lung cancer. Cancer Med (2021) 10(7):2216–31. doi: 10.1002/cam4.3649 PMC7982619 33655698

[33] Xiong L Cai Y Zhou X Dai P Wei Y Zhao J . Optimum immunotherapy method according to PD-L1 expression in advanced lung cancer: a network meta-analysis. Future Oncol (London England). (2022) 18(7):883–96. doi: 10.2217/fon-2021-1217 34825576

[34] Acheampong E Abed A Morici M Bowyer S Amanuel B Lin W . Tumour PD-L1 expression in small-cell lung cancer: A systematic review and meta-analysis. Cells. (2020) 9(11):E2393. doi: 10.3390/cells9112393 PMC7693331 33142852

[35] Yu H Boyle TA Zhou C Rimm DL Hirsch FR . PD-L1 expression in lung cancer. J Thorac Oncol (2016) 11(7):964–75. doi: 10.1016/j.jtho.2016.04.014 PMC5353357 27117833

[36] He M Zheng T Zhang X Peng Y Jiang X Huang Y . First-line treatment options for advanced non-small cell lung cancer patients with PD-L1 ≥ 50%: a systematic review and network meta-analysis. Cancer immunology immunotherapy (2022) 71(6):1345–55. doi: 10.1007/s00262-021-03089-x PMC10991470 34657171

[37] Gay CM Stewart CA Park EM Diao L Groves SM Heeke S . Patterns of transcription factor programs and immune pathway activation define four major subtypes of SCLC with distinct therapeutic vulnerabilities. Cancer Cell (2021) 39(3):346–360.e347. doi: 10.1016/j.ccell 33482121 PMC8143037

[38] Zhu L Xu Y Kang S Lin B Zhang C You Z . Quantification-promoted discovery of glycosylated exosomal PD-L1 as a potential tumor biomarker. Small Methods (2022) 6(9):e2200549. doi: 10.1002/smtd.202200549 35810463

[39] Zhang J Zhu Y Guan M Liu Y Lv M Zhang C . Isolation of circulating exosomes and identification of exosomal PD-L1 for predicting immunotherapy response. Nanoscale. (2022) 14(25):8995–9003. doi: 10.1039/d2nr00829g 35700522

[40] Ayala-Mar S Donoso-Quezada J González-Valdez J . Clinical implications of exosomal PD-L1 in cancer immunotherapy. J Immunol Res (2021) 2021:8839978. doi: 10.1155/2021/8839978 33628854 PMC7886511

[41] Ouyang Y Liu W Zhang N Yang X Li J Long S . Prognostic significance of programmed cell death-ligand 1 expression on circulating tumor cells in various cancers: A systematic review and meta-analysis. Cancer Med (2021) 10(20):7021–39. doi: 10.1002/cam4.4236 PMC8525108 34423578

[42] Dall'Olio FG Gelsomino F Conci N Marcolin L De Giglio A Grilli G . PD-L1 expression in circulating tumor cells as a promising prognostic biomarker in advanced non-small-cell lung cancer treated with immune checkpoint inhibitors. Clin Lung cancer. (2021) 22(5):423–31. doi: 10.1016/j.cllc.2021.03.005 33849808

[43] Spiliotaki M Neophytou CM Vogazianos P Stylianou I Gregoriou G Constantinou AI . Dynamic monitoring of PD-L1 and Ki67 in circulating tumor cells of metastatic non-small cell lung cancer patients treated with pembrolizumab. Mol Oncol (2022). doi: 10.1002/1878-0261.13317 PMC10158784 36177552

[44] Schulz C Gandara D Berardo CG Rosenthal R Foo J Morel C . Comparative efficacy of second- and subsequent-line treatments for metastatic NSCLC: A fractional polynomials network meta-analysis of cancer immunotherapies. Clin Lung cancer. (2019) 20(6):451–460.e455. doi: 10.1016/j.cllc.2019.06.017 31375454

[45] Almutairi AR Alkhatib N Martin J Babiker HM Garland LL McBride A . Comparative efficacy and safety of immunotherapies targeting the PD-1/PD-L1 pathway for previously treated advanced non-small cell lung cancer: A Bayesian network meta-analysis. Crit Rev oncology/hematology. (2019) 142:16–25. doi: 10.1016/j.critrevonc.2019.07.004 31326706

[46] Huang Y Fan H Li N Du J . Risk of immune-related pneumonitis for PD1/PD-L1 inhibitors: Systematic review and network meta-analysis. Cancer Med (2019) 8(5):2664–74. doi: 10.1002/cam4.2104 PMC6536966 30950194

[47] Chen X Zhang Z Hou X Zhang Y Zhou T Liu J . Immune-related pneumonitis associated with immune checkpoint inhibitors in lung cancer: A network meta-analysis. J immunotherapy cancer. (2020) 8(2):e001170. doi: 10.1136/jitc-2020-001170 PMC7462235 32863271

